# The Metabolic Syndrome: Prevalence, Associated Factors, and Impact on Survival among Older Persons in Rural Bangladesh

**DOI:** 10.1371/journal.pone.0020259

**Published:** 2011-06-15

**Authors:** Masuma Akter Khanam, Chengxuan Qiu, Wietze Lindeboom, Peter Kim Streatfield, Zarina Nahar Kabir, Åke Wahlin

**Affiliations:** 1 ICDDR,B Knowledge for Global Lifesaving Solutions, Dhaka, Bangladesh; 2 Department of Neurobiology, Care Sciences and Society (NVS), Aging Research Centre, Karolinska Institutet, Stockholm, Sweden; 3 Division of Nursing, NVS, Karolinska Institutet, Stockholm, Sweden; 4 Department of Psychology, Stockholm University, Stockholm, Sweden; Innsbruck Medical University, Austria

## Abstract

**Objectives:**

To describe the prevalence of the metabolic syndrome (MetS) among older persons in rural Bangladesh, to investigate whether the prevalence varies by age, sex, literacy, marital status, nutritional status and socio-economic status, and to assess the impact of MetS on survival.

**Methods:**

The study consisted of 456 persons who were aged ≥60 years living in a rural area of Bangladesh during July 2003–March 2004. Data were collected through interview, clinical examination, and laboratory tests, and their survival status until 30^th^ June 2009 was ascertained through the Matlab surveillance system. We defined MetS following the NCEP ATP III criteria, with minor modifications, i.e., presence of any three of the following: hypertension (BP ≥130/85 mm Hg); random blood glucose (RBG) level ≥7.0 mmol/L; hyper-triglyceridemia (≥2.28 mmol/L); low level of HDL-cholesterol (<1.04 mmol/L for men and <1.29 mmol/L for women); and BMI ≥25.0 kg/m^2^. Data were analysed with logistic regressions for the influential factors of MetS, and with Cox models for the association of MetS with the survival status.

**Findings:**

The overall prevalence of MetS was 19.5%, 20.8% in women, and 18.0% in men. Asset-index and nutritional status were independently associated with MetS. During 4.93 years of follow-up, 18.2% died. In the presence of high RBG, MetS has a significant negative effect on survival (69.4% vs 95.2%, log rank p = 0.02).

**Conclusion:**

This study highlights the importance of the metabolic syndrome in rural Bangladesh. Our findings suggest that there is a need for screening programmes involving the metabolic syndrome to prevent diabetes and cardiovascular diseases.

## Introduction

The metabolic syndrome (MetS) is a constellation of obesity, hyperglycaemia, decreased high density lipoprotein (HDL), increased triglyceride, and high blood pressure [1]. The MetS, as a driver of current epidemics of diabetes [2] and cardiovascular diseases (CVD) [3], has become a major challenge to public health around the world. The association of MetS with myocardial infarction (MI), stroke, and diabetes, has been extensively documented [4] and CVD is the major cause of death in the developing world [5]. Importantly, about 80% of the global burden of CVD related death occurs in low- and middle-income countries [6].

World-wide, the prevalence of MetS ranges from 10% to 50% [7]. The detection, prevention, and treatment of the MetS components should become an important approach for the reduction of the cardiovascular disease burden in the general population [8]. Hence, the identification of the population at risk is of utmost importance.

In recent years, several studies [9]–[15] have shown the increasing importance of MetS in low-income countries, but few studies have focused on rural populations. In this population-based study of persons aged 60 years and above living in rural Bangladesh, we seek (1) to determine the prevalence of MetS, (2) to examine factors related to MetS, and (3) to assess the impact of MetS on survival.

## Methods

### Participants

Study participants were from the “Poverty and Health in Ageing”, a collaborative project between Ageing Research Centre at Karolinska Institutet, Sweden and ICDDR,B, Bangladesh. This is a cross-sectional study of elderly persons aged 60 years or more, conducted in Matlab, a rural sub-district of Bangladesh. Since 1966, icddr,b has maintained a Health and Demographic Surveillance System (HDSS) in this area which is divided into seven blocks and covers a population of approximately 220,000 across 142 villages, where regular update of all vital events is maintained. For this study, we selected two blocks, which are nearest to the main Matlab hospital.

### Ethics Statement

Ethical approval was received from both Karolinska Institutet, Sweden and Institutional Review Board (IRB) ICDDR,B Bangladesh. Information about the study was provided to and informed consent was obtained from all participants.

Of the 850 randomly selected older persons from the two purposively selected blocks, 63 died before data collection, 38 refused to participate, 11 migrated, 93 could not be reached, 18 were registered twice in the surveillance database, and 2 persons were found to be under 60 years of age. Thus, 625 respondents were interviewed in their homes, of whom 473 participated in the clinical examination. Complete information for the current study was available for 456 persons, slightly more than 50% of the original sample. The non-participants (n = 169) were more likely to be women, older and with poor socio-economic status, but their health profile did not differ from the respondents [16].

### Data collection

Between July 2003 and March 2004, data were collected in two sessions on two separate days. Data on demographics, socio-economic status, and self-reported morbidity were collected during a home interview by trained interviewers. Clinical examinations were conducted at the local ICDDR,B health centre by physicians and blood samples were taken for laboratory analyses.

### Definition of the metabolic syndrome

We define the MetS following the criteria proposed by the Third Report of the National Cholesterol Education Program Expert Panel on the Detection, Evaluation, and Treatment of High Blood Cholesterol in Adults (NCEP ATP III) [17]. The NCEP ATP III criteria comprise 3 or more of the following: (1) fasting plasma glucose level of at least 110 mg/dL (6.1 mmol/L); (2) serum triglyceride level of at least 150 mg/dL (1.7 mmol/l) for men; (3) serum high-density lipoprotein (HDL) cholesterol level lower than 40 mg/dL (1.04 mmol/L) for men and lower than 50 mg/dL (1.29 mmol/L) for women; (4) Blood Pressure of at least 130/85 mm Hg or controlled with antihypertensive treatment; and/or (5) waist circumference of more than 102 cm (Adult Treatment Plan III 2001). The NCEP ATP III definition does not specify that any particular component be present and implies that if a person has several risk factors, even if they are not very severe, their joint contribution can still markedly increase the risk of coronary heart disease.

In this study, the MetS is defined as having any 3 of the following: 1) high blood glucose ≥7.0 mmol/L; 2) hypertriglyceridemia (triglyceride level ≥2.28 mmol/L); 3) low level of HDL cholesterol (<1.04 mmol/L for men and <1.29 mmol/L for women); 4) BMI ≥25.0 kg/m^2^and 5) hypertension (systolic BP ≥130 mm Hg or diastolic BP ≥85 mm Hg).

We used non-fasting blood sample for biochemical analysis because it was not possible to request the fasting state of the elderly people. Further we used a cut-off ≥200 mg/dL (≥2.28 mmol/L) to define high triglyceride, which is in line with other studies [18].

Literacy was defined as the ability to read and write in Bangla language. Asset index is used as proxy for socio-economic status of the respondents. Asset index data was collected separately from the surveillance database of HDSS of ICDDR,B. The asset index is based on household assets and housing characteristics, including such as bed, mattress, quilt, cooking pots, watch, chair, clothing cabinet, radio, television, bicycle, boat, cows and electricity. Using a variable reduction technique, these assets and characteristics were combined into a single variable. After ranking this variable from low to high, households were divided into 3 equally sized groups, the poverty tertiles. Details about the calculation of the asset index can be found elsewhere [19]. Marital status was categorized as married or single. Divorced, widows, widowers and never married persons were included in the single group, and the category married represents currently married persons. Nutritional status was assessed using the Mini Nutritional Assessment (MNA) [20]. The calculation of the nutritional status has been described in detail previously [16]. For this study we grouped the ‘undernourished’ and ‘at risk of malnutrition’ into ‘not well-nourished’.

Height and weight were measured with the participant barefoot and lightly dressed. Body mass index was calculated as weight (kg) divided by height squared (m^2^). Blood pressure was measured by sphygmomanometer, after taking a 5-min rest, and with standard procedure. The sitting-position measurements were used in this study. Plasma lipids (high-density lipoprotein [HDL] cholesterol and triglycerides) and glucose were measured using standard enzymatic methods. Random blood glucose was measured by an ACCU-CHECK glucometer.

### Mortality data

Data on survival status of all participants until 30^th^ June 2009 are from the routine surveillance program of HDSU of ICDDR,B, in which ICD-10 is used to clarify the cause of death (ICD-10, WHO).

### Data analyses

Descriptive analyses included mean and standard deviations (SD) for continuous variables. Prevalence and frequencies are expressed in terms of percentage. Categorical variables were compared by chi-square statistic. Logistic regression was used to examine factors related to MetS. Kaplan-Meyer survival analysis was used to examine the relationship of MetS, as well as its various components, with survival up to five years. Also, Cox model was applied for estimating the hazard ratio for mortality by MetS. All analyses were performed using the statistical software SPSS Statistics version 11.5 for Windows.

## Results

Table 1 presents the characteristics of study participants, The mean age of the 456 participants was 69 years (SD 6.8; range 60–92), and 55% were female.

**Table 1:** Characteristics of the study population (n = 456) by status of the metabolic syndrome.

*Characteristics*	*Number of subjects, n (%)*	*The metabolic syndrome*		
		*Present*	*Absent*	*p-value*
*Age group, years*				
60–69	273 (59.9)	56 (62.9)	217 (59.1)	
70+	183 (40.1)	33 (37.1)	150 (40.9)	0.298
*Sex*				
Men	206 (45.2)	37 (41.6)	169 (46.0)	
Women	250 (54.8)	52 (58.4)	198 (54.0)	0.261
*Literacy*				
Illiterate	272 (60.0)	48 (54.5)	224 (61.4)	
Literate	181 (40.0)	40 (45.5)	141 (38.6)	0.146
*Marital status*				
Single	194 (42.5)	38 (42.7)	156 (42.5)	
Married	262 (57.5)	51 (57.3)	211 (57.5)	0.533
*Asset Index*				
Lowest tertile	143 (33.1)	14 (16.7)	129 (37.1)	
Middle tertile	145 (33.6)	37 (44.0)	108 (31.0)	
Highest tertile	144 (33.3)	33 (39.3)	111 (31.9)	0.001
*Smoking*				
No	272 (59.8)	60 (67.4)	212 (57.9)	
Yes	183 (40.2)	29 (32.6)	154 (42.1)	0.064
*Tobacco leaf*				
No	134 (30.4)	32 (37.2)	102 (28.7)	
Yes	307 (69.6)	54 (62.8)	253 (71.3)	0.082
*Nutritional status*				
Not well nourished	388 (87.6)	63 (74.1)	325 (90.8)	
Well nourished	55 (12.4)	22 (25.9)	33 (9.2)	<0.001

The prevalence of low HDL was 98.2%, in combination with the other components, this leaves only 0.4% (two individuals) without any MetS risk component. All five risk components were present in 0.9% of the participants (data not shown), and high blood pressure was prevalent 49.8% (Table 2). The prevalence of the MetS components did not differ significantly between men and women, except high triglycerides where women had a higher prevalence than men (p = 0.005) (Table 2). The overall prevalence of MetS was 19.5%, slightly higher prevalence in women (20.8%) compared to men (18.0%) (p = 0.26) (Table 3). The prevalence of MetS did not change consistently with increasing age (Table 3).

**Table 2:** Prevalence of different components of the metabolic syndrome by sex.

*Components*	*Total, n (%)*	*Men, n (%)*	*Women, n (%)*	*p-value* *
*Low HDL*	448 (98.2)	204 (99.0)	244 (97.6)	0.215
*High blood pressure*	227 (49.8)	102 (49.5)	125 (50.0)	0.496
*High Random blood glucose*	60 (13.2)	25 (12.1)	35 (14.0)	0.329
*High triglyceride*	89 (19.5)	29 (14.1)	60 (24.0)	0.005
*High BMI*	24 (5.3%)	7 (3.4)	17 (6.8)	0.078

*p-value is for comparison between men and women.

**Table 3:** Prevalence of the metabolic syndrome by age and gender.

	Men			Women			Total		
Age group, years	N	n	Prevalence (%)	N	n	Prevalence (%)	N	n	Prevalence (%)
60–64	60	12	32.4	79	15	28.8	139	27	30.3
65–69	65	11	29.7	69	18	34.6	134	29	32.6
70–74	41	7	18.9	43	8	15.4	84	15	16.9
≥75	40	7	18.9	59	11	21.2	99	18	20.2
Total	206	37	18.0	250	52	20.8	456	89	19.5

Asset index and nutritional status were found to be independently associated with MetS (Table 4).

**Table 4:** Unadjusted and adjusted odds ratios (OR) and 95% confidence intervals (CI) of Metabolic Syndrome: Results from logistic regression analyses.

	*Metabolic syndrome*	
	*Unadjusted OR (95% CI)*	*Adjusted OR (95% CI)* a
*Age group, years*		
60–69	1.00 (Ref)	1.00 (Ref)
70+	0.85 (0.53–1.38)	0.84 (0.48–1.46)
*Sex*		
Men	1.00 (Ref.)	1.00 (Ref.)
Women	1.20 (0.75–1.92)	0.90 (0.32–2.53)
*Literacy* *		
Illiterate	1.00 (Ref.)	1.00 (Ref.)
Literate	1.32 (0.83–2.11)	1.09 (0.61–1.95)
*Marital Status* *		
Single	1.00 (Ref.)	1.00 (Ref.)
Married	0.99 (0.62–1.59)	0.96 (0.47–1.97)
*Asset index* #		
Lowest tertile	1.0 (Ref.)	1.00 (Ref.)
Middle tertile	3.16 (1.62–6.14)	2.95 (1.41–6.15)
Highest tertile	2.74 (1.40–5.38)	2.78 (1.31–5.89)
Smoking		
No	1.00 (Ref.)	1.00 (Ref.)
Yes	0.67 (0.41–1.09)	0.58 (0.23–1.46)
Tobacco leaf		
No	1.00 (Ref.)	1.0 (Ref.)
Yes	0.68 (0.42–1.12)	0.81 (0.47–1.42)
Nutritional status§		
Not well-nourished	1.0 (Ref.)	1.0 (Ref.)
Well-nourished	3.44 (1.88–6.29)	3.05 (1.58–5.90)

OR = odds ratio; CI = confidence interval.

*Data on literacy and marital status were missing for 3 participants.

# Data on asset index was missing for 24 persons.

§ Data on Nutritional status was missing for 13 persons.

a ORs (95% CIs) were derived from the model that included age, sex, literacy, marital status, asset index, smoking, tobacco leaf and nutritional status.

**Figure 1:**
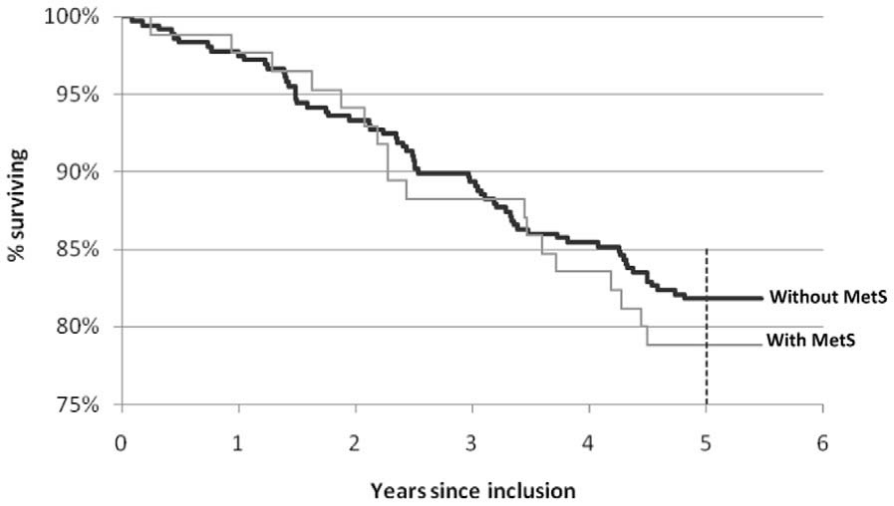
Survival curve of the elderly people by metabolic syndrome, up to 5 years.

Of the 456 participants, 83 (18.2%) died during the follow-up of 4.93 years. During this time period 14 (3.1%) left the study area and were considered lost to follow up. Analyses of the survival status of this population suggested that those with MetS had a slightly lower survival rate (78.8%) compared to those without MetS (81.8%). This difference was however not statistically significant (log rank p = 0.55, df = 1) (Figure 1). Cox regression analyses suggested that the hazard ratio of death related to MetS was 1.17 (95% CI 0.70–1.98). The major cause of death in this sample was circulatory system related (61.4%); cerebro-vascular diseases being the most prevalent cause followed by ischemic heart disease and then hypertensive diseases. Deaths due to the circulatory system constituted 66.7% of the total number of deaths in the MetS group and 60% of the deaths in the non MetS group (not statistically significant).

**Figure 2:**
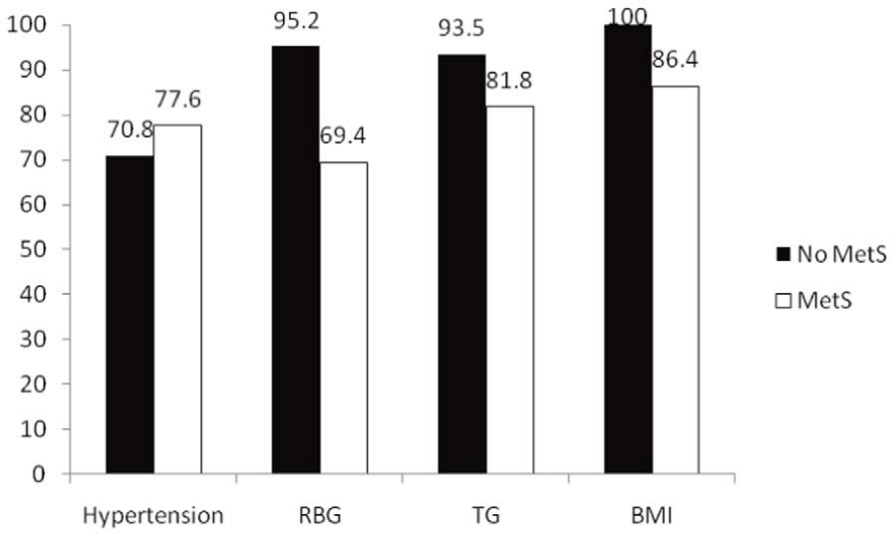
Five-years survival related to MetS by different components of MetS. (* p<0.05). RBG = Random Blood Glucose, TG = Triglyceride, BMI = Boby Mass Index.

In further analysis, we found that, in the presence of high RBG, MetS has a significant negative effect on survival (69.4% vs 95.2%, log rank p = 0.02, df = 1). Survival with the other individual components triglyceride, high blood pressure and high BMI did not reveal any significant differences (Figure 2).

## Discussion

In this study we have found the overall prevalence of MetS was 19.5%, 20.8% in women, and 18.0% in men. Asset-index and nutritional status were independently associated with MetS. During 4.93 years of follow-up, 18.2% died. Cox regression analysis suggested that the hazard ratio of death related to MetS was 1.17 (95% CI 0.70–1.98). In the presence of high RBG, MetS has a significant negative effect on survival (69.4% vs 95.2%, log rank p = 0.02)

This study was based on a random sample of elderly individuals living in the community in Matlab, a rural area of Bangladesh. The prevalence of MetS was 19.5%, consistent with findings from other studies, e.g., the Botnia study in Finland and Sweden 22.6% [21] and 12% in the Risk Factors and Life Expectancy Study (RIFLE) study in Italy [22]. Recent studies from developing countries reported various prevalence rates depending on the criteria used in defining the MetS. In Iran, using the NCEP criteria, prevalence of MetS was estimated to be between 24% and 30%, depending on sex [23]. In India, 25% of subjects had the MetS by IDF criteria [24], another study reported the prevalence by NCEP criteria as 9.3% in rural central India [25]. In Seychelles, an island country of African region, according to the ATP, WHO and IDF definitions, the prevalence of MetS was, respectively, 24.0%, 25.0%, 25.1% in men and 32.2%, 24.6%, 35.4% in women [26]. In the semi-rural areas of Turkey the prevalence of MetS was 27.6% by NCEP criteria [27]. There is a scarcity of data regarding the prevalence of MetS in Bangladesh. However, in a study conducted amongst rural Bangladeshi women (≥18 years), the prevalence was low (<3%) [28]. Another prevalence study conducted on clinic based hypertensive patients (20–79 years) found prevalence of 64% [29]. This is the first population based data regarding prevalence of MetS among older persons of rural Bangladesh. This study underscores the importance of metabolic syndrome in rural Bangladesh as one in every five elderly subjects is affected by the syndrome. In our study we have found a slightly higher, although not significant, prevalence rate of MetS among the women, similar to some studies [30], [22] and contrary to other [21].

The prevalence of high blood pressure was 50% and 49% among women and men respectively, which is a finding similar to figures reported in other studies [21], [31]. We have found a very high prevalence of low HDL cholesterol in this population (98.2%). Evidence exists that Asian Indians have low HDL [32], [33], and the level of HDL 2b, the most protective component of HDL is low in >90% of Asian Indians [34]. In a Bangladeshi sample of hypertensive women aged 20–79 years, the prevalence of low HDL cholesterol was more than 90% [29]. Another study conducted on Bangladeshi rural women aged 18 years and above reported the prevalence of low HDL cholesterol as 65% [28]. Among the South Asians, the prevalence of low HDL is higher among the people of Bangladesh (52%), nearly double to that of the Pakistanis (30%) and three times higher than that of the Indians (17%). In an African sample, the prevalence of dyslipidemia (TG >150 mg% or HDL cholesterol <40 mg%) was 92% among the persons with MetS [31].

In studying the determinants, we found that nutritional status and asset index were significantly associated with MetS. The absence of significant differences by age groups is contrasting prevalence studies performed in the west [22], [35]. A likely reason is that people in Bangladesh with this syndrome develop coronary heart diseases and die at a younger age. Furthermore, it is evident that people from this region (South East Asia) are prone to developing CHD at a younger age, often before the age of 40 years [36].

The interesting relationship between socioeconomic status and MetS is sometimes mediated by behavioural factors, which are related to both MetS, and to socioeconomic status [37]. Contrary to some [38]–[40], but in line with others [41], we didn't find any association between smoking and MetS, neither between the use of tobacco leaf and MetS. Indeed, smoking tends to be associated with a decreased odds ratio of MetS. Because this analysis is based on cross-sectional data, it is likely that survival bias may, at least partly, account for the result, especially when both smoking and MetS were associated with high mortality. We found instead independent relationships of MetS with socioeconomic status, assessed by asset index; the respondents belonged to the middle and the highest tertiles are at significantly higher risk of having MetS, compared to the lowest, hence the poorest one third of the respondents. Factors associated with this relationship appear to be, paradoxically, things which many people would regard as improvements in their lives. They include higher consumption of meat; the respondents who belonged to the highest tertile were approximately four times more likely to consume meat daily compared to the people who belonged to the lowest, hence the poorest tertile in this study population, and milk consumption increased from 47% among the lowest tertile to 69% among the respondents belonged to the highest tertile. Well nourished people were at higher risk of MetS in this study. Evidence exists that better nutritional status is associated with higher socioeconomic status [16], [42]–[43]. We also found a significant correlation between asset index and nutritional status (r = 0.149) (p = 0.002) in our study population. It was not possible to find the causal direction of relationships of asset index, well nourishment, and MetS, but the study confirms the positive associations of more wealth and well nourishment with the risk of MetS. More studies are warranted to explore a causal relationship of higher prevalence of MetS and better socio-economic status.

Multivariate logistic regression analyses were conducted to examine the association of independent variables (age, sex, literacy, marital status, smoking, use of tobacco leaf, nutrition and asset index) with each component of the MetS. Among the variables studied, association of age with hypertension and triglyceride was in opposite direction. The risk of high blood pressure is associated with higher age, whereas risk is reduced with higher age for high triglyceride. The overall association of nutrition and asset index with metabolic syndrome was mostly due to their association with high triglyceride and high BMI.

Overall, MetS was not associated with 5-year survival, although our analysis suggests a potential impact of MetS on poor survival among individuals with high RBG. Due to small sample size and relatively short follow up time the study lacks power to show significant difference in survival rates.

Further analyses were performed to explore if the results differ with changing the cutoff of components. We didn't find any difference in results. The prevalence of MetS ranged between 14.5 *ceteris paribus*, changing the cutoff for blood pressure from ≥130 mm Hg to ≥140 mm Hg and 22.8 *ceteris paribus*, changing the cutoff for TG from ≥2.28 to ≥1.7 mmol/L. Nutrition and asset index remained statistically significant as before. The association of well nourishment with metabolic syndrome remained significant with a variation of odds ratio from 2.02 to 4.49 when changing the cutoffs of different components. Only when we change the cutoff for blood pressure from ≥130 mm Hg to ≥140 mm Hg, the wealthiest tertile become insignificant. Survival rates ranged between 75% to 79.4%.

Several limitations of the study should be mentioned. The cross-sectional design requires that caution should be taken before making causal interpretations on the basis of the findings. Waist circumference was not available, instead we used BMI as a proxy for abdominal adiposity in conformity with other studies that have used BMI to replace waist circumference [9], [44]–[48]. Although measurements were based on non-fasting blood samples, which particularly affects triglyceride and glucose levels, other studies using non fasting measurement have found the predictivity for CHD comparable to studies that have used fasting measurements [18]. Non-fasting triglyceride levels are on the average 20% to 30% higher than fasting levels. We have used a cut-off ≥200 mg/dL (≥2.28 mmol/L) to define high triglyceride, which is in line with other relevant studies [18]. The WHO criteria for diagnosis of the MetS is more complex because it takes into consideration microalbuminuria, plasma insulin level. NCEP definition of metabolic syndrome does not specify that any particular component be present. The NCEP criteria imply that if one has many risk factors, even if they are not very severe, their joint contribution can still markedly increase the risk of CHD.

Murray and Lopez (1996) predicted that CVD will increase in low- and middle-income countries, and that it will be the leading cause of death and disability worldwide by 2020 [49]. The economic and social costs of this burden will be great, particularly because many developing nations are still grappling with poverty-related diseases such as malnutrition, infectious diseases, and poor health care facilities. It is desirable to identify the warning signs of the MetS, thereby facilitating early education and intervention among these individuals. Additional prospective long-term studies are required to identify the combination of components of the MetS that identify more precisely persons who will develop type 2 diabetes and/or cardiovascular morbidity. Our findings highlight the importance of MetS and may be useful for designing future preventive actions against diabetes and cardiovascular diseases.

## References

[1] Magliano DJ, Shaw JE, Zimmet PZ (2006). How to best define the metabolic syndrome.. Ann Med.

[2] Zimmet P, Alberti KG, Shaw J (2001). Global and societal implications of the diabetes epidemic.. Nature.

[3] Grundy SM, Brewer B, Cleeman JI, Smith SC, Lenfant C (2004). Definition of metabolic syndrome: report of the National Heart, Lung, and Blood Institute/American Heart Association conference on scientific issues related to definition.. Circulation.

[4] Cassells HB, Haffner SM (2006). The metabolic syndrome: risk factors and management.. J Cardiovasc Nurs.

[5] Ezzati M, Hoorn SV, Lawes CM, Leach R, James WP (2005). Rethinking the “diseases of affluence” paradigm: global patterns of nutritional risks in relation to economic development.. PLoS Med.

[6] Gaziano TA, Opie LH, Weinstein MC (2006). Cardiovascular disease prevention with a multidrug regimen in the developing world: a cost-effective analysis.. Lancet.

[7] Cameron AJ, Shaw JE, Zimmet PZ (2004). The Metabolic Syndrome: Prevalence in Worldwide Populations.. Endocrinol Metab Clin North Am.

[8] Eckel RH, Grundy SM, Zimmet PZ (2005). The metabolic syndrome.. Lancet.

[9] Shiwaku K, Nogi A, Kitajima K, Anuurad E, Enkhmaa B (2005). Prevalence of the Metabolic Syndrome using the Modified ATP III Definitions for Workers in Japan, Korea and Mongolia.. J Occup Health.

[10] Kasliwal RR, Bansal M, Agarwal V, Dandona P, Mehrotra R (2004). Metabolic Syndrome and Cardiovascular Risk Factors Among the Affluent Asian Indians Living in India.. Metabo Syndr Rel Dis.

[11] Deepa M, Farooq S, Datta M, Deepa R, Mohan V (2007). Prevalence of metabolic syndrome using WHO, ATPIII and IDF definitions in Asian Indians: the Chennai Urban Rural Epidemiology Study (CURES-34).. Diabetes Metab Res Rev.

[12] Cheung BMY (2006). The metabolic syndrome in China.. Br J Clin Pharmacol.

[13] Enas EA, Mohan V, Deepa M, Farooq S, Pazhoor S (2007). The metabolic syndrome and dyslipidemia among Asian Indians: a population with high rates of diabetes and premature coronary artery disease.. J Cardiometab Syndr.

[14] Pan A, Franco OH, Ye J, Demark-Wahnefried W, Ye X (2008). Soy protein intake has sex-specific effects on the risk of metabolic syndrome in middle-aged and elderly Chinese.. J Nutr.

[15] Tan CE, Ma S, Waid D, Chew SK, Tai ES (2004). Can We Apply the National Cholesterol Education Program Adult Treatment Panel Definition of the Metabolic Syndrome to Asians?. Diabetes Care.

[16] Kabir ZN, Ferdous T, Cederholm T, Khanam MA, Streatfied K (2006). Mini Nutritional Assessment of rural elderly people in Bangladesh: the impact of demographic, socio-economic and health factors.. Public Health Nutr.

[17] Expert Panel on Detection, Evaluation, and Treatment of High Blood Cholesterol in Adults (2001). Executive summary of the third report of the National Cholesterol Education Program (NCEP) Expert Panel on Detection, Evaluation, and Treatment of High Blood Cholesterol in Adults (Adult Treatment Panel III).. JAMA.

[18] Wannamethee SG, Shaper AG, Lennon L, Morris RW (2005). Metabolic Syndrome vs Framingham Risk Score for Prediction of Coronary Heart Disease, Stroke, and Type 2 Diabetes Mellitus.. Arch Intern Med.

[19] Razzaque A, Streatfield P, Gwatkin D (2007). Does health intervention improve socioeconomic inequalities of neonatal, infant and child mortality? Evidence from Matlab, Bangladesh.. Int J Equity Health.

[20] Guigoz Y, Vellas B, Garry PJ (1994). Mini Nutritional Assessment: a practical tool for grading the nutritional state of elderly patients.. Facts Res Gerontol Suppl.

[21] Gause-Nilsson I, Gherman S, Kumar Dey D, Kennerfalk A, Steen B (2006). Prevalence of metabolic syndrome in an elderly Swedish population.. Acta Diabetol.

[22] Trevisan M, Liu J, Bahsas FB, Menotti A (1998). Syndrome X and mortality: a population-based study. Risk Factor and Life Expectancy Research Group.. Am J Epidemiol.

[23] Esteghamati A, Khalilzadeh O, Rashidi A, Meysamie A, Haghazali M (2009). Association between physical activity and metabolic syndrome in Iranian adults: national surveillance of risk factors of noncommunicable diseases (SuRFNCD-2007).. Metabolism.

[24] Sachdev HP, Osmond C, Fall CH, Lakshmy R, Ramji S (2009). Predicting adult metabolic syndrome from childhood body mass index: follow-up of the New Delhi birth cohort.. Arch Dis Child.

[25] Kamble P, Deshmukh PR, Garg N (2010). Metabolic syndrome in adult population of rural Wardha, central India.. Indian J Med Res.

[26] Kelliny C, William J, Riesen W, Paccaud F, Bovet P (2008). Metabolic syndrome according to different definitions in a rapidly developing country of the African region.. Cardiovasc Diabetol.

[27] Arikan I, Metintas S, Kalyoncu C, Colak O, Arikan U (2009). Evaluation of metabolic syndrome prevalence in semi-rural areas of Central Anatolia, Turkey.. Saudi Med J.

[28] Zaman MM, Ahmed J, Choudhury SR, Numan SM, Islam MS (2006). Prevalence of Metabolic Syndrome in Rural Bangladeshi Women.. Diabetes Care.

[29] Siddique MA, Sultan MAU, Haque KMHSS, Zaman MM, Ahmed CM (2008). Clustering of metabolic factors among the patients with essential hypertension.. Bangladesh Med Res Counc Bull.

[30] Scuteri A, Najjar SS, Morrell CH, Lakatta EG (2005). Cardiovascular Health Study. The metabolic syndrome in older individuals: prevalence and prediction of cardiovascular events: the Cardiovascular Health Study.. Diabetes Care.

[31] Isezuo SA, Ezunu E (2005). Demographic and clinical correlates of metabolic syndrome in Native African type-2 diabetic patients.. J Natl Med Assoc.

[32] Bhalodkar NC, Blum S, Rana T, Bhalodkar A, Kitchappa R (2004). Comparison of levels of large and small high-density lipoprotein cholesterol in Asian Indian men compared with Caucasian men in the Framingham Offspring Study.. Am J Cardiol.

[33] Enas EA, Mohan V, Deepa M, Farooq S, Pazhoor S (2007). The Metabolic Syndrome and Dyslipidemia Among Asian Indians: A Population With High Rates of Diabetes and Premature Coronary Artery Disease.. J Cardiometab Syndr.

[34] Superko HR, Enas EA, Kotha P, Bhat NK, Garrett B (2005). High-density lipoprotein subclass distribution in individuals of Asian Indian descent: the National Asian Indian Heart Disease Project.. Prev Cardiol.

[35] Isomaa B, Almgren P, Tuomi T, Forsen B, Lahti K (2001). Cardiovascular morbidity and mortality associated with the metabolic syndrome.. Diabetes Care.

[36] Enas EA, Yusuf S, Mehta J (1996). Meeting of the International Working Group on Coronary Artery Disease in South Asians.. Indian Heart J.

[37] Paek KW, Chun KH, Jin KN, Lee KS (2006). Do Health Behaviors Moderate the Effect of Socioeconomic Status on Metabolic Syndrome?. Ann Epidemiol.

[38] Ramsay SE, Whincup PH, Morris R, Lennon L, Wannamethee SG (2008). Is socioeconomic position related to the prevalence of metabolic syndrome? influence of social class across the life course in a population-based study of older men.. Diabetes Care.

[39] Oh SW, Yoon YS, Lee ES, Kim WK, Park Lee S (2005). Association between cigarette smoking and metabolic syndrome: the Korea National Health and Nutrition Examination Survey.. Diabetes Care.

[40] Masulli M, Riccardi G, Galasso R, Vaccaro O (2006). Relationship between smoking habits and the features of the metabolic syndrome in a non-diabetic population.. Nutr Metab Cardiovasc Dis.

[41] Park HS, Kim SM, Lee JS, Lee J, Han JH (2007). Prevalence and trends of metabolic syndrome in Korea: Korean National Health and Nutrition Survey 1998–2001.. Diabetes Obes Metab.

[42] Dapi LN, Janlert U, Nouedoui C, Stenlund H, Håglin L (2009). Socioeconomic and gender differences in adolescents' nutritional status in urban Cameroon, Africa.. Nutr Res.

[43] Ferdous T, Kabir ZN, Wahlin Å, Streatfield K, Cederholm T (2009). The multidimensional background of malnutrition among rural older individuals in Bangladesh – a challenge for the Millennium Development Goal.. Public Health Nutr.

[44] Morimoto A, Nishimura R, Suzuki N, Matsudaira T, Taki K (2008). Low prevalence of metabolic syndrome and its components in rural Japan.. Tohoku J Exp Med.

[45] Sattar N, Gaw A, Scherbakova O, Ford I, O'Reilly DS (2003). Metabolic syndrome with and without C-reactive protein as a predictor of coronary heart disease and diabetes in the West of Scotland Coronary Prevention Study.. Circulation.

[46] Malik S, Wong ND, Franklin SS (2004). Impact of the metabolic syndrome on mortality from coronary heart disease, cardiovascular disease, and all causes in United States Adults.. Circulation.

[47] Ridker PM, Buring JE, Cook NR, Rifai N (2003). C-reactive protein, the metabolic syndrome, and risk of incident cardiovascular events: an 8 year follow-up of 14 719 initially healthy American women.. Circulation.

[48] Aguilar-Salinas CA, Rojas R, Gómez-Pérez FJ, Valles V, Ríos-Torres JM (2004). High prevalence of metabolic syndrome in Mexico.. Arch Med Res.

[49] Murray CJL, Lopez AD (1996). The global burden of disease: A comprehensive assessment of mortality and disability from diseases, injuries and risk factors in 1990 and projected to 2020.

